# Fractional Vegetation Cover Estimation Based on an Improved Selective Endmember Spectral Mixture Model

**DOI:** 10.1371/journal.pone.0124608

**Published:** 2015-04-23

**Authors:** Ying Li, Hong Wang, Xiao Bing Li

**Affiliations:** 1 State Key Laboratory of Earth Surface Processes and Resource Ecology, College of Resources Science and Technology, Beijing Normal University, Beijing, China; 2 CERI eco Technology Company Limited, Beijing, China; University of Chinese Academy of Sciences, CHINA

## Abstract

Vegetation is an important part of ecosystem and estimation of fractional vegetation cover is of significant meaning to monitoring of vegetation growth in a certain region. With Landsat TM images and HJ-1B images as data source, an improved selective endmember linear spectral mixture model (SELSMM) was put forward in this research to estimate the fractional vegetation cover in Huangfuchuan watershed in China. We compared the result with the vegetation coverage estimated with linear spectral mixture model (LSMM) and conducted accuracy test on the two results with field survey data to study the effectiveness of different models in estimation of vegetation coverage. Results indicated that: (1) the RMSE of the estimation result of SELSMM based on TM images is the lowest, which is 0.044. The RMSEs of the estimation results of LSMM based on TM images, SELSMM based on HJ-1B images and LSMM based on HJ-1B images are respectively 0.052, 0.077 and 0.082, which are all higher than that of SELSMM based on TM images; (2) the R^2^ of SELSMM based on TM images, LSMM based on TM images, SELSMM based on HJ-1B images and LSMM based on HJ-1B images are respectively 0.668, 0.531, 0.342 and 0.336. Among these models, SELSMM based on TM images has the highest estimation accuracy and also the highest correlation with measured vegetation coverage. Of the two methods tested, SELSMM is superior to LSMM in estimation of vegetation coverage and it is also better at unmixing mixed pixels of TM images than pixels of HJ-1B images. So, the SELSMM based on TM images is comparatively accurate and reliable in the research of regional fractional vegetation cover estimation.

## Introduction

Vegetation is the comprehensive result of the long-term interaction of landform, hydrology, soil, climate variability and human activities and its distribution, composition and development are closely related with environment condition, especially climate condition [1–3]. As an important parameter reflecting horizontal coverage degree of vegetation on land surface, fractional vegetation cover is the percentage of the vertical projection of vegetation (branch, stem and leaves) in the statistic area of land surface [4,5]. Vegetation coverage is an important quantitative information measuring the vegetation coverage status on the ground, which is also a sensitive indicator of evaluating land degradation and desertification [6] and it is also a controlling factor of universal soil loss equation (USLE) and revised universal soil loss equation (RUSLE), numerical climate model and hydro-ecological model [7–10]. Deriving regional land surface fractional vegetation cover and its change information is of significant meaning to discover the responses of ecosystem and the rules of spatial change, discuss the driving factors of such response, analyze and evaluate regional eco-environment under the influences of global change[11,12].

Currently, there are two methods of getting vegetation coverage, field measurement and remote sensing monitoring method. The former is a traditional method of obtaining vegetation coverage which includes visual estimation method, sampling method and instrument method according to different measuring modes [13] and plays an major role in survey of land surface vegetation. It is routinely widely applied because of its high accuracy. However, limited by time, weather and regional condition, this method is expensive and labor-intensive and can only provide the information of vegetation changes, composition and distribution in particular areas, so it is inappropriate to depict vegetation coverage from macro-scale regions [14–16]. With the development of remote sensing technique in monitoring of vegetation coverage, field measurement is gradually no longer the dominant method, but it is still very important in research and application of estimation of vegetation coverage on surface or near surface. For example, in estimation of vegetation coverage with space remote sensing technique, field survey data are usually used for the sensitivity analysis of the result of remote sensing estimation model [17–20].

The development of remote sensing technique provides measurement of vegetation coverage with a new direction. The characteristics of large scale and periodic detection with remote sensing data make it possible to obtain vegetation coverage and its dynamic change in a large area and it has been widely applied [21–22]. In most studies, there are three basic approaches to estimate fractional vegetation cover from remote sensing data: regression model, vegetation index method and mixed pixel unmixing model [23, 24]. Pixel unmixing model is mainly spectral mixture model, which extracts vegetation coverage with linear or nonlinear mixture model. Linear mixture model is based on the assumption that each pixel in an image can be decomposed into a linear combination of different components and the photon which reaches sensor only acts with one component. Because of the simple and practical feature, this model is widely applied when there are low spectral resolution and spatial resolution in images [25, 26] and it plays a crucial role in estimation fractional vegetation cover in arid and semi-arid regions [27–28]. Previous studies illustrate that to some extents, LSMM is superior to other remote sensing inversion approaches when estimating fractional vegetation cover of mono temporal data [29]. In the spectral unmixing procedure, all endmembers in an image are used in traditional LSMM to each pixel, but in fact, for low- or moderate- spatial resolution imageries, most mixed pixels are just composed of a small part of the whole endmembers set, so application of related endmembers for unmixing mixed pixel will correspondingly increase the accuracy. Pixel Pure Index (PPI) endmember extraction algorithm is a more successful and wider application method, which is based on the minimum noise transform to extract spectral information of various types of surface features. Cong Hao et al.[30] first developed a spectral mixture analysis approach based on selective endmembers. Through calculating the response value to measure the spectral similarity between the actual pixel and the reference endmembers, a series of appropriate endmembers can be extracted dynamically. It can be guaranteed an endmembers with high spectral similarity is selected which has been applied in several studies [31–34]. However, the response value of traditional SELSMM is defined as the endmember proportion within each pixel. The sum of the response values between all the endmember spectrums and pixel spectrum is set to 1. This assumption is lack of enough mathematical or physical basis.

According to the theory that different pixels used different set of endmembers to decompose spectrum, through calculating the response value between reference endmember spectrum and actual pixel spectrum, we judged the similarity degree of the two spectra. Taken the response value of a reference endmember as the spectral contribution value of the reference endmember to actual pixel so that it can participate in unmixing of mixed pixel, we developed a new selective endmember linear spectral mixture model (SELSMM). Selecting the Huangfuchuan watershed in China as the study site, we estimated the fractional vegetation cover from Landsat TM image and HJ-1B image. The accuracy of results was assessed through field measured coverage acquired in the same period. The feasibility of applying the two spectral mixing models to estimate vegetation coverage in study site based on different images was also discussed.

## Data and Methods

### Study area

**Fig 1:**
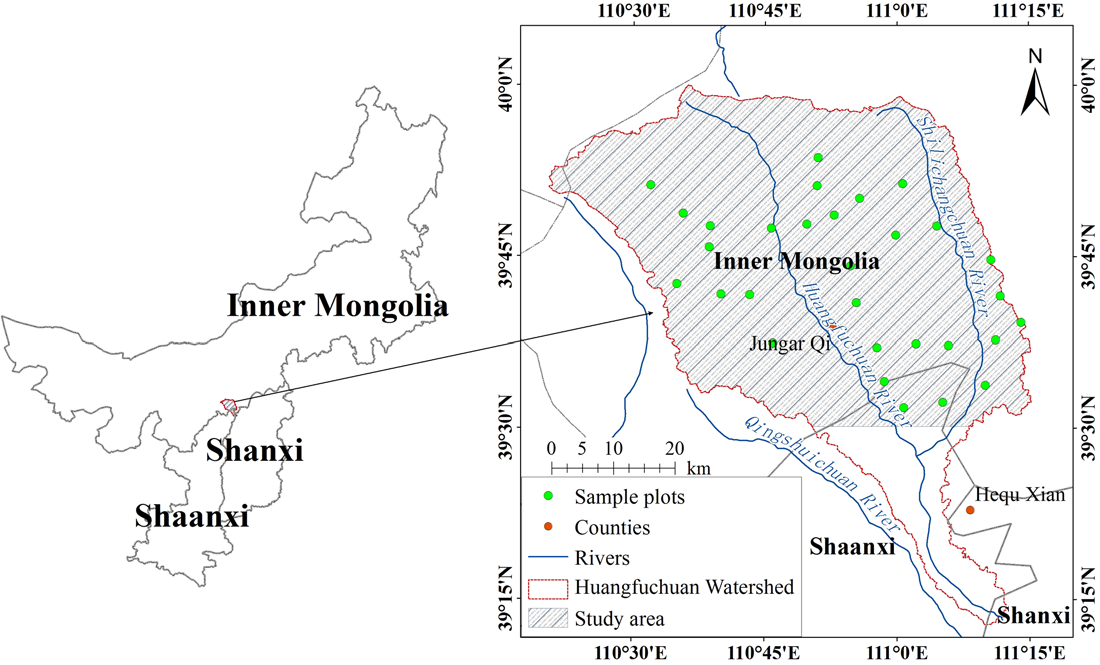
Location of study site.

In this study, Huangfuchuan watershed in northern China was selected as the study site (Fig 1). As a main branch of the middle reach of the Yellow River, Huangfuchuan River originates from the district in Aobaoliang of southern Dalad Banner and Dianpangou of northwestern Jungar Banner in Inner Mongolia. It flows into the Yellow River in Batuping of Fugu County in Shannxi Province. Huangfuchuan watershed covers the zones between longitude 110.3°E and 111.2°E and latitude 39.2°N and 39.9°N with total area of 3342km^2^ throughout Ordos Plateau and Loess Plateau. It has a semi-arid continental monsoon climate with average annual temperature of 6.2°C and average annual rainfall of 368.7mm. Rainfall is main concentrated in summer, more than 80% of which happens between June and September. The average potential evaporation is about 2040mm per year. Strong and frequent wind occurs in winter and spring with average annual wind speed ranging from 2 m·s^-1^ to 3 m·s^-1^ and 10 to 30 windy days.

The study site is located in temperate semi-arid grassland kastanozem zone and the soil is mainly composed of kastanozem with feldspathic sandstone as soil parent material, aeolian sandy soil, the loessal soil and loess with loess as soil parent material. The native vegetation is typical steppe, such as *Stipabungean* steppe. Now, the main macrophanerophytes are artificial *Pinus tabulaeformis* forest, artificial *Poplar* forest and some surviving natural coniferous woodlands, such as *Platyclaclus*, *Juniperus communis*, *Juniperus formosana* and *Pinus tabulaeformis*. Shrubbery is characterized by *Salix cheilophila*, *Fructus Hippophae*, *Caraganaintermediakuanget H*. *D*. *Fn*, etc. The native *Stipabungean* steppe has been replaced by natural vegetation such as *Thymusserpyllum* steppe. The herbaceous community is dominated by *Thymusserpyllum*, *Stipabungean*, *Heteropappusaltaicu*, *Cleistogenessquarros*, etc.

### Data and preprocessing

#### Field survey data

Field survey data were used as the training data to calibrate statistical models describing relationship between remote sensing parameters and vegetation cover. We positioned the sample plots with GPS and designed sample plots referring to topographic map and vegetation chart. The size of samples plots matched with the spatial resolution of remote sensing images perfectly, which was 30×30m^2^. We collected 24 shrub sample plots and 6 grassland sample plots with 3 quadrats at each sample plot, including 90 quadrats in total. Specific permission was not required for all the sample plots and for the sampling activities. The field study activities did not involve any endangered or protected species.

In grassland sample plots, we selected a 1×1m^2^ quadrat randomly and took photos vertically with a digital camera. After geometric correction, enhancement processing, color space transformation and classification, the grassland fractional cover of each photo was extracted. For each plot, we calculated the arithmetic mean of vegetation cover from all quadrats to acquire the fractional cover. Coverage data in quadrats were then transformed to data in sample plots on surface. In shrub sample plots, we utilized line-intercept method, which involved two objectives: ①put three survey lines with 30m long at each sample plot, pulled the measure tape tight along the survey lines, calculated the fractional cover by dividing the length of tape that was intercepted by plants by the total tape length; ②selected three grassland quadrats of 1×1m^2^, collected grassland coverage using the same protocol as used in grassland sample plots. Sum of the two was the vegetation coverage in shrub sample plots.

#### Remote Sensing Data Acquisition and Pre-processing

A Landsat-5 TM image and a HJ-1B image were acquired in this study. We acquired cloud-free TM image with a relatively high spatial resolution (30m) was on August 7, 2011. The image provided by the small satellites constellation of environment and disaster monitoring and forecasting in China was obtained on July 30, 2011 with the CCD camera fixed on HJ-1B satellite and its spatial resolution was 30m.

The images were Level 1T products with primary precision and terrain correction. Geometric correction was necessary for TM image and HJ-1B image by different radiometric correction models according to the topographic map of study site.

(1) For TM image, we converted the DN (digital number) value to radiance using Eq (1) to eliminate the sunshine difference in multispectral images.

$$L=\frac{L_{\max}-L_{\min}}{255}\times DN+L_{\min}$$ (1)

where *L*_*max*_ and *L*_*min*_ are the spectrum radiance values when the DN values a 255 and 1 respectively, which can be obtained from Internet (http://landsat.usgs.gov/science_L5_cpf.php).

We input the parameters obtained from atmospheric correction which was conducted with 6S model into atmospheric correction Eq (2) and got the atmospheric correction results image.

$$acr_{i}=\frac{xa_{i}\times L_{i}-xb_{i}}{1+xc_{i}\times (xa_{i}\times L_{i}-xb_{i})}$$ (2)

where *i* = 1,2,3,4,5,7, which represents the wave band of TM images; *L* is the radiance after radiometric calibration; *xa*, *xb* and *xc* are atmospheric correction parameters in 6S model.

(2) For HJ-1B image, satellite image was calibrated with the radiometric calibration equation which could transform the DN image of HJ-1B satellite acquired from China Centre for Resources Satellite Data and Application.

$$L=DN/a+L_{0}$$ (3)

where *L* is radiance value, 1/*a* is the gain of absolute calibration coefficient, *L*_0_ is offset. The unit of transformed radiance value is *W*·*m* ^-2^·*sr* ^-1^·*um* ^-1^

Conducted by radiative transfer model codes of MODTRAN4+(Moderate Resolution Atmospheric Transmission),we used the FLAASH atmospheric correction module in ENVI to compensate for the effects of atmosphere, sunlight, etc. on surface features reflection, so as to restore the surface reflectance of surface features from images and then obtained the atmospheric corrected image.

### Methods

#### Processing steps of the study

**Fig 2:**
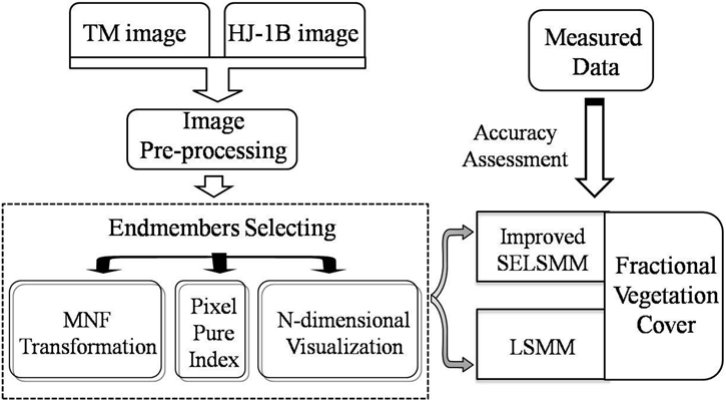
Major processing steps in study.

Based on TM image and HJ-1B image, we estimated the fractional vegetation cover in study site with LSMM and improved SELSMM respectively. The major processing is shown in Fig 2. The study incorporated three sections: 1) select endmembers from images which have been processed with PPI method to ensure that the pixels participating in spectral unmixing are purer; 2) extract vegetation coverage information in study site from TM image and HJ-1B image with improved SELSMM and compare it with the estimation result of LSMM; and 3) quantify the accuracy of the results estimated with different models based on the two kinds of images using field measured coverage in the same period.

#### Spectral Unmixing

*Selection of Endmembers*: In the procedure of spectral unmixing, it must be sure that the selected endmembers can represent the spectral information of all land cover components truly and objectively, so that the result will have a high accuracy. The number of endmembers is constrained by the dimensionality of satellite imagery. More endmembers can interpret more spectral heterogeneity and then improve applicability of the model. However, too many endmembers will make model more sensitive to endmember selection and then the universality of model will be reduced. So, it is critical to determine the balance between number of endmembers and the overall optimization of the model. In this study, we selected endmembers by calculating PP). First, we minimum noise fraction (MNF) transformed the images to reduce the spectral dimensionality so that the major information and noise could be abstracted and the redundancy of data and correlation among wave bands could be reduced. On such basis, we could determine the candidate endmembers through analyzing PPI. We connect MNF transformation with PPI using the n-Dimensional Visualization tool to apply n-dimensional visualization analysis and extract the spectral information of all components [34]. In this study, we just need fractional vegetation cover, so it is unnecessary to classify non-vegetation features. V-I-S model (Vegetation-Impervious Surface-Soil model) was adopted to identify the surface components and spectral reflectivity curve of endmembers for spectral unmixing [34].

*Linear Spectral Mixture*: Model. Each pixel in a remote sensing image can be composed of a finite number of dominant components which have relatively contribution to the spectral information collected by remote sensing sensor. The remote sensing information of every pixel can be unmixed into several components and those components constitute the remote sensing information of the pixel through a linear combination. Therefore, linear unmixing can be conducted on remote sensing information to build a pixel mixture model for estimation of vegetation coverage. LSMM is the most common model for unmixing of mixed pixel, which is defined as that the reflectance of a pixel on a certain spectral band (brightness value) is the linear combination of reflectance of the basic components (Endmember) with their proportions in total area of the pixel as the weight coefficients [35]. The model (Eq (4)) and its constraint conditions (Eq (5) and Eq (6)) are shown below:

$$R_{Li}=\overset{n}{\underset{j=1}{\sum }}C_{Lj}\alpha_{ij}+\varepsilon_{Li}$$ (4)

$$\overset{n}{\underset{j=1}{\sum }}\alpha_{ij}=1$$ (5)

$$0\leq \alpha_{ij}\leq 1$$ (6)

where *R*_*Li*_ is the spectral reflectance of pixel *i* on wave band *L*; *α*_*ij*_ is the proportional value of the basic component *j* of pixel *I*; *C*_*Lj*_ is the spectral reflectivity of basic component *j* on wave band *L*; *n* is the number of basic components of pixel *i*; *ε*_*Li*_ is the residual not explained by the linear model.

When calculating the proportions of basic components of image pixels, fully constrained linear spectral mixture model (FCLSMM) was chosen to make the fractional vegetation cover estimation with LSMM more accurate and reliable, that was to calculate *α*_*ij*_ with the constraints of minimum *ε*_*Li*_, nonnegative *α*_*ij*_ and a sum of 1 for *α*_*ij*_.

*Improved Selective Endmember Linear Spectral Mixture Model*: We built the SELSMM based on the theory that different pixels used different set of endmembers to decompose spectrum. The key of model was how to determine the endmembers comprised in each pixel. Through calculating the response value between reference endmember spectrum and actual pixel spectrum, we can judge the similarity degree of the two spectra. Taken the response value of a reference endmember as the spectral contribution value of the reference endmember to actual pixel so that it can participate in unmixing of mixed pixel, a SELSMM can be built 33]. The higher the response value between reference endmember spectrum and pixel spectrum is, the more similar the two spectras are and the more proportion the component which corresponds to the reference endmember has in the pixel. Therefore, an improved algorithm for selective endmember is developed. Normalization processing was conducted on the response value *x*_*i*_ between the endmember spectrum for comparing and spectrum of a certain pixel firstly. These response values will be mapped to the range from 0 to 1 through the proportional relationship of $\frac{x_{i}}{\overset{m}{\underset{i=1}{\sum }}x_{i}}$ (*x*_*i*_ is response value between the endmember spectrum for comparing and spectrum of a certain pixel; *m* is the number of response values which participate in similarity degree comparison). Finally the proportion was proposed as the contribution value of endmember *i* to the pixel.

With the new calculating mean of contribution value, linear spectral unmixing can be conducted on selective endmember and then the estimation accuracy of the model can be enhanced.

(1)Through calculating the response value between the actual pixel spectrum and reference endmember spectrum, the spectral similarity between the two spectra can be derived, so it can be guaranteed that the reference endmember spectrum which is highly similar to the actual pixel spectrum will be selected. Response coefficient is obtained by dividing the covariance of the two spectra by the product of their variances. The spectrum response coefficient of a pixel can be represented as:

$$r_{ij}=\frac{m\overset{m}{\underset{L=1}{\sum }}C_{\text{L}j}{\text{R}}_{\text{L}i}-\overset{m}{\underset{L=1}{\sum }}C_{\text{L}j}\overset{m}{\underset{L=1}{\sum }}{\text{R}}_{\text{L}i}}{\sqrt{\left[m\overset{m}{\underset{L=1}{\sum }}C_{\text{L}j}^{2}-{\left(m\overset{m}{\underset{L=1}{\sum }}C_{\text{L}j}\right)}^{2}\right]\left[m\overset{m}{\underset{L=1}{\sum }}{\text{R}}_{\text{L}i}^{2}-{\left(m\overset{m}{\underset{L=1}{\sum }}{\text{R}}_{\text{L}i}\right)}^{2}\right]}}$$ (7)

where *C*_*Lj*_ is the spectrum of reference endmember *j* on wave band *L*; *R*_*Li*_ is the spectrum of pixel *i* on wave band *L*; *m* is the number of spectral wave bands.

(2) Calculating and comparing the response values between different referential endmembers spectra and actual pixels spectra with Eq (8), the maximum response value (denoted *r*_*max*_) and corresponding endmember spectrum vector (denoted ***A***_***max***_) can be obtained. As the reference endmember spectrum which is most similar to the pixel, ***A***_***max***_ can be taken as the preferred endmember.

Setting a parameter *X*_*j*_ as the contribution value of a reference endmember ***A***_***j***_ to mixed pixel ***R***_***i***_, the contribution of reference endmember ***A***_***max***_ to mixed pixel ***R***_***max***_ can be represented as *X*_*max*_ and the contribution of residual endmember (denoted ***R***_***Re***_) to ***R***_***i***_ can be represented as:

$${\text{R}}_{\text{Re}}={\text{R}}_{\text{i}}-X_{\text{max}}•{\text{R}}_{\text{max}}$$ (8)

Combining the response value *r*_*j*_ of reference endmember to mixed pixel, *X*_*j*_ can be represented as:

$$X_{j}=\frac{r_{j}}{\overset{n}{\underset{j=1}{\sum }}r_{j}}$$ (9)

$$\overset{n}{\underset{j=1}{\sum }}X_{j}=1$$ (10)

Then, the contribution of reference endmember ***A***_***max***_ to mixed pixel ***R***_***max***_ should be:

$$X_{\max}=\frac{r_{\max}}{\overset{n}{\underset{j=1}{\sum }}r_{j}}$$ (11)

Replacing the *X*_*max*_ in Eq (8) with Eq (11), residual endmember ***R***_***Re***_ can be represented as:

$${\text{R}}_{\text{Re}}={\text{R}}_{\text{i}}-\frac{r_{\max}}{\overset{n}{\underset{j=1}{\sum }}r_{j}}\times {\text{R}}_{\text{max}}$$ (12)

A series of experiments indicated that as the selected endmember spectrum vectors are non-orthogonal, the identifying procedure may encounter end when ***R***_***Re***_ meet the terminal conditions even after one iteration. So, we added an adjustment coefficient *η* and adjusted Eq (12) into the following pattern:

$${\text{R}}_{\text{Re}}={\text{R}}_{\text{i}}-\eta \times \frac{r_{\max}}{\overset{n}{\underset{j=1}{\sum }}r_{j}}\times {\text{R}}_{\text{max}}$$ (13)

where *η* ranges from 0 to 1 and to some extent it influences the number of endmembers *n* within each pixel: when *η* is too large, the identifying procedure may encounter with just a few times of iteration of a pixel, while if *η* is too small, all endmembers will participate in the calculation and calculation of spectrum response value will become meaningless. According to many experiments, we demonstrated that 0.35 and 0.65 would be the appropriate values of *η* for Landsat TM images and HJ-1B images respectively.

(3) Replacing the ***R***_***i***_ in Eq (8) with ***R***_***Re***_ and conducting iteration on Eq (7) to Eq (13), the iteration will stop when a component of ***R***_***Re***_ becomes negative or there is only a small variation of Δ***R*** (Eq (14)) and then the number of endmembers (*n*) in the pixel and corresponding endmember spectrum can be determined.

$$\Delta \text{R}={\text{R}}_{\text{Re}}^{\text{k}+1}-{\text{R}}_{\text{Re}}^{\text{k}}$$ (14)

where ${\text{R}}_{\text{Re}}^{\text{k}+1}$ and ${\text{R}}_{\text{Re}}^{\text{k}}$ are respectively the residual pixel spectrum after iterations of k+1 times and k times.

With above method, the number of endmembers in each pixel and corresponding endmember spectrum vectors can be determined. Together with FCLSMM, the content of every component of each pixel in an image can be obtained.

#### Fractional vegetation cover estimation

**Fig 3:**
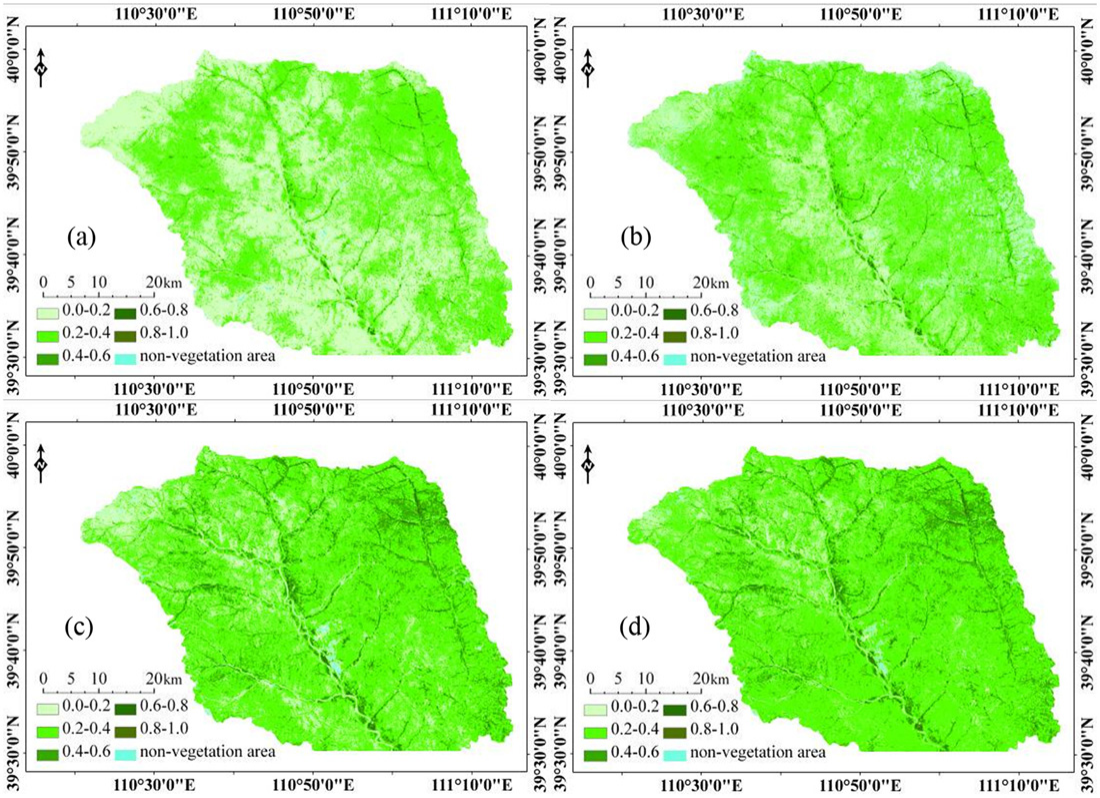
Estimation results of fractional vegetation cover. ((a) fractional vegetation cover estimated with LSMM based on TM image; (b) fractional vegetation cover estimated with SELSMM based on TM image; (c) fractional vegetation cover with LSMM based on HJ-1B image; (4) fractional vegetation cover SELSMM based on HJ-1B image.)

We unmixed the mixed pixels of TM image and HJ-1B image respectively with LSMM and SELSMM and obtained the fractional vegetation cover in study site. To compare the estimated results based on different models and images facilitate, the fractional vegetation cover is classified into six categories, namely 0.0~0.2, 0.2~0.4, 0.4~0.6, 0.6~0.8, 0.8~1.0 and non-vegetation (Fig 3). We calculated the area of each category and obtained their percentages of the total area in study area (Table 1). Fig 3 shows that in study site, the region with fractional vegetation cover ranging from 0.0 to 0.2 is mostly in loess covered terrain with high altitude while the region with fractional vegetation cover ranging from 0.2 to 0.4 is the widest, which matches with the actual vegetation coverage situation in the study site. The region with fractional vegetation cover ranging from 0.4 to 0.6 distributed in the plain area along the river is mostly covered by farmland vegetation and artificial shrub, where the fractional vegetation cover is high. There is little region with fractional vegetation cover larger than 0.8. Region of non-vegetation is mainly water area such as rivers, lakes, reservoirs, etc. and area with impervious surface such as mines, cities, etc.

**Table 1:** Proportions of the area of different fractional vegetation cover categories which is estimated based on different models and different images.

Category	Proportion (*P*, %)			
	LSMM^TM^	SELSMM^TM^	LSMM^HJ-1B^	SELSMM^HJ-1B^
0.0–0.2	49.21	30.71	15.05	9.29
0.2–0.4	49.10	62.78	71.54	76.46
0.4–0.6	1.55	3.35	12.23	13.14
0.6–0.8	0.13	0.22	0.81	0.79
0.8–1.0	0.01	0.01	0.08	0.15
**non-vegetation**	0.00	2.94	0.29	0.15

The proportions listed in the table are the proportions of the area of different fractional vegetation cover categories in the total area of the study area. LSMM^TM^, SELSMM^TM^, LSMM^HJ-1B^ and SELSMM^HJ-1B^ respectively indicate LSMM based on TM image, SELSMM based on TM image, LSMM based on HJ-1B image and SELSMM based on HJ-1B image.

**Fig 4:**
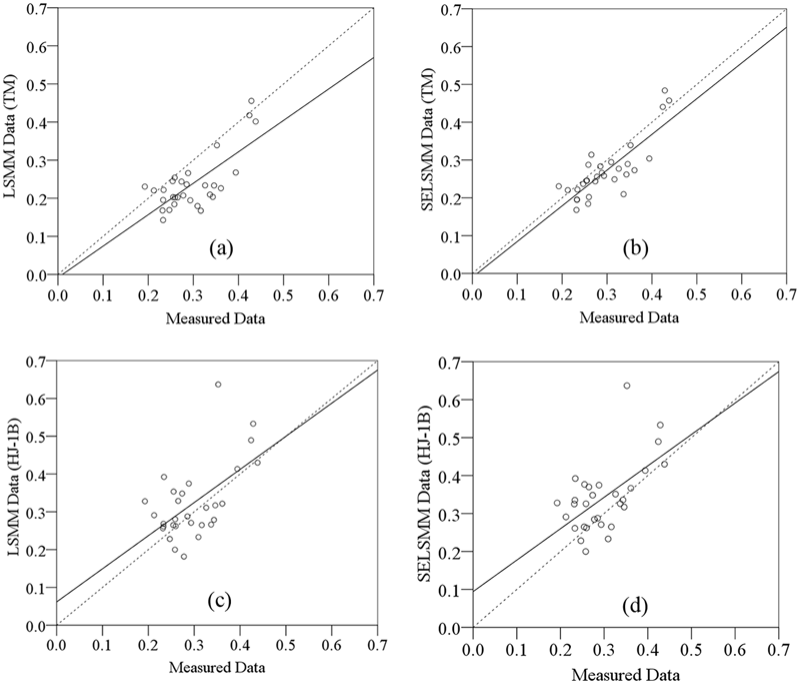
Relationship between estimated fractional vegetation cover with models and field survey data. ((a) result of linear regression analysis between vegetation coverage which is estimated with LSMM based on TM image and measured coverage; (b) result of linear regression analysis between vegetation coverage which is estimated with SELSMM based on TM image and measured coverage; (c) result of linear regression analysis between vegetation coverage which is estimated with LSMM based on HJ-1B image and measured coverage; (d) result of linear regression analysis between vegetation coverage which is estimated with SELSMM based on HJ-1B image and measured coverage.)

The accuracy of fractional vegetation cover estimated by models was assessed through the field survey data. The size of random samples for collection of field measured coverage data is 30×30m^2^ to match the spatial scale of field survey data to the spatial resolution of images. We extracted the estimated coverage corresponding to the spatial position of field survey data to conducted linear regression analysis with measured coverage data. The result shows that all optimal regression equations pass t-test of 0.05, which meet the statistical demand (p<0.05) with a significant correlation between estimated and field coverage data (Table 2). The samples do not show clear segregation by vegetation and converge to the 1:1 straight line (Fig 4). Therefore, fractional vegetation cover in study site can be extracted with LSMM and SELSMM based on TM image and HJ-1B image. For the unmixing results of a certain model, the overall accuracy of pixel unmixing based on TM image is higher than the results based on HJ-1B image. The RMSEs of the former are smaller than the latter; In the linear regression models which are built between the fractional vegetation cover estimated with a certain remote sensing model and field measured coverage, the R^2^ of the estimation result based on TM image is larger than that based on HJ-1B image.

**Table 2:** Accuracy of the fractional vegetation cover estimated with different models based on different images.

Images	Models	Accuracy		
		RMSE	R^2^	P
**TM image**	LSMM	0.052	0.531	0.000
	SELSMM	0.044	0.668	0.000
**HJ-1B image**	LSMM	0.082	0.336	0.001
	SELSMM	0.077	0.342	0.001

To compare the difference between the estimated coverage and measured coverage, we selected root mean square error (RMSE) criterion and coefficient of determination (R^2^) to evaluate estimation accuracy and the results are shown in Table 2. RMSE is used to assess the overall accuracy of mixture models and the coefficient of determination reflects the fitting degree between estimated and field survey data.

$$RMSE=\sqrt{\frac{1}{N}\overset{N}{\underset{i=1}{\sum }}{\left(f_{i}^{'}-f_{i}\right)}^{2}}$$ (15)

$$R^{2}=\frac{\overset{N}{\underset{i=1}{\sum }}(f_{i}^{'}-\bar{f})}{\overset{N}{\underset{i=1}{\sum }}{\left(f_{i}-\bar{f}\right)}^{2}}$$ (16)

where $f_{i}^{'}$ is the fractional vegetation cover estimated with models and *f*_i_ is the corresponding measured coverage; $\bar{f}$ is the average value of estimated fractional vegetation cover; *N* is the number of samples for accuracy assessment.

## Discussion

### Comparison of Fractional Vegetation Cover Estimated with LSMM and SELSMM

Based on the fractional vegetation cover estimated with the two models and TM image and HJ-1B image, we get the area proportions of different fractional vegetation cover categories (Table 1). For TM image and HJ-1B image, the regions with the estimated vegetation coverage from 0.2 to 0.4 occupies the largest area proportion (except for the coverage estimated with LSMM based on TM image), which matches to the result of field survey. For the same kind of images: (1) the area proportion of the regions with fractional vegetation cover ranging from 0.0 to 0.2 estimated with LSMM is larger than that estimated with SELSMM; (2) however, opposite result is achieved for the area proportions of the regions with vegetation coverage in the ranges of 0.2~0.4, 0.4~0.6 and 0.8~1.0, that is to say the fractional vegetation cover estimated with SELSMM enjoys a larger proportion of area in above ranges. LSMM for all pixels with the same ingredients endmember performs spectral decomposition, while SELSMM for each pixel using different components of endmember, namely endmember composition changes with components of pixel and a corresponding change in the number of endmember thus effectively improve the estimation accuracy.

Comparison of the estimation accuracy of the two models shows that the RMSE of the estimation result of LSMM is larger than that of SELSMM, both of which are smaller than 0.100. Concerning unmixing result of a certain kind of images, the overall accuracy of LSMM in mixed pixel unmixing is smaller than that of SELSMM, with RMSE-values of 0.052(LSMM/TM), 0.044(SELSMM/TM), 0.082(LSMM/HJ-1B), 0.077(SELSMM/HJ-1B). For R^2^—values of the two kinds of images, the estimation result of LSMM has a smaller R^2^ (0.531 for LSMM based on TM image, 0.668 for SELSMM based on TM image, 0.336 for LSMM based on HJ-1B image, 0.342 for SELSMM based on HJ-1B image), which indicates that comparing with the estimation result of LSMM, there is a higher correlation between the fractional vegetation cover estimated with SELSMM and field measured coverage. On the whole, in the study site, the fractional vegetation cover estimated with SELSMM is superior to the result estimated with LSMM.

### Comparison of Fractional Vegetation Coverage Estimated Based on TM Image and HJ-1B Image

From Table 1, we derive the area proportions of different fractional vegetation cover categories based on TM image and HJ-1B image. For the fractional vegetation cover estimated with the same model: (1) the area proportion of regions with fractional vegetation cover between 0.0 and 0.2 which is estimated based on TM image is smaller than that estimated based on HJ-1B image; (2) An opposite result is achieved for the area proportion of regions with fractional vegetation cover larger than 0.2, that is to say the fractional vegetation cover estimated based on HJ-1B image occupies a larger proportion of area in above ranges.

Comparison of the estimation accuracy of the two kinds of images shows that (Table 2): There is a higher correlation between the vegetation coverage obtained from spectral unmixing models based on TM image and field measured coverage comparing to what obtained based on HJ-1B image. Therefore, in the study site, the fractional vegetation cover obtained by spectral unmixing models based on TM image is more accurate.

## Conclusions

Moderate resolution remote sensing images, such as Landsat TM and HJ-1B, represent an attractive source of information for estimating the fractional vegetation cover in arid and semi-arid regions. LSMM is widely used in various studies. In this study, a new SELSMM has been put forward, which uses reference endmembers of each pixel in an image to enhance the unmixing accuracy. With Landsat TM image and HJ-1B image as data sources, we unmixed the mixed pixels respectively with LSMM and SELSMM and obtained the fractional vegetation cover within area of Huangfuchuan watershed. In addition, we verified the estimation accuracy of the two models with field measured data of the same period and conducted comparative analysis. The results indicate that the estimation result of SELSMM based on TM image enjoys the highest accuracy while the estimation result of LSMM based on HJ-1B image enjoys the lowest accuracy. Generally, the estimation results of SELSMM are more accurate than that of LSMM. Additionally, TM image is more suitable for mixed pixel unmixing. As there is comparatively less spectral information in HJ-1B image, it is difficult to unmix pixel with models based on such images very accurately with the same spatial resolution.

For comparison of LSMM and SELSMM, choosing a universal set of reference endmembers that will be required for spectral mixture analysis, is often not feasible. The algorithm of selective endmember can dynamically define the actual number of endmembers for spectral unmixing according to the spectra information in each pixel. This method possibly eliminated the adverse impacts by excluding irrelated endmembers, consequently, estimation of fractional vegetation cover in study site will be more accurate. In this study, processing is conducted on the response value between reference endmembers spectrum and actual pixel spectrum and then the normalized response value is taken as the contribution value of reference endmembers to the pixel. In addition, in order to determine the number of endmembers and endmember spectrum for unmixing mixed pixels, an adjustment coefficient is introduced. The accuracy of linear spectral unmixing can be enhanced to some extents by this method. Nonetheless, there are still some problems to be resolved. For example, whether it is reasonable to conduct spectral unmixing with the same set of average endmember spectrum in the whole area and whether the identification of endmembers which are actually comprised in each mixed pixel will be influenced in this method.
